# Dysfunctional missense variant of *OAT10/SLC22A13* decreases gout risk and serum uric acid levels

**DOI:** 10.1136/annrheumdis-2019-216044

**Published:** 2019-11-28

**Authors:** Toshihide Higashino, Keito Morimoto, Hirofumi Nakaoka, Yu Toyoda, Yusuke Kawamura, Seiko Shimizu, Takahiro Nakamura, Kazuyoshi Hosomichi, Akiyoshi Nakayama, Keiko Ooyama, Hiroshi Ooyama, Toru Shimizu, Miki Ueno, Toshimitsu Ito, Takashi Tamura, Mariko Naito, Hiroshi Nakashima, Makoto Kawaguchi, Mikiya Takao, Yosuke Kawai, Naoki Osada, Kimiyoshi Ichida, Ken Yamamoto, Hiroshi Suzuki, Nariyoshi Shinomiya, Ituro Inoue, Tappei Takada, Hirotaka Matsuo

**Affiliations:** 1 Department of Integrative Physiology and Bio-Nano Medicine, National Defense Medical College, Tokorozawa, Japan; 2 Graduate School of Information Science and Technology, Hokkaido University, Sapporo, Japan; 3 Department of Pharmacy, The University of Tokyo Hospital, Tokyo, Japan; 4 Division of Human Genetics, Department of Integrated Genetics, National Institute of Genetics, Mishima, Japan; 5 Laboratory for Mathematics, National Defense Medical College, Tokorozawa, Japan; 6 Department of Bioinformatics and Genomics, Graduate School of Advanced Preventive Medical Sciences, Kanazawa University, Kanazawa, Japan; 7 Ryougoku East Gate Clinic, Tokyo, Japan; 8 Midorigaoka Hospital, Takatsuki, Japan; 9 Division of Nursing, National Defense Medical College, Tokorozawa, Japan; 10 Department of Internal Medicine, Self-Defense Forces Central Hospital, Tokyo, Japan; 11 Department of Preventive Medicine, Nagoya University Graduate School of Medicine, Nagoya, Japan; 12 Department of Preventive Medicine and Public Health, National Defense Medical College, Tokorozawa, Japan; 13 Genome Medical Science Project (Toyama), National Center for Global Health and Medicine, Tokyo, Japan; 14 Department of Pathophysiology, Tokyo University of Pharmacy and Life Sciences, Tokyo, Japan; 15 Department of Medical Biochemistry, Kurume University School of Medicine, Kurume, Japan

**Keywords:** gout, epidemiology, gene polymorphism

Organic anion transporter 10 (OAT10), also known as SLC22A13, has hitherto been identified as a urate transporter by *in vitro* analyses.^1^ Despite the reported expression of OAT10 on the apical membrane of the renal proximal tubular cells,^1^ the physiological impact of OAT10 on urate handling in humans remains to be elucidated. Accumulating evidence suggests that functional variants of already-characterised, physiologically important urate transporters—URAT1/SLC22A12, GLUT9/SLC2A9, BCRP/ABCG2 and NPT1/SLC17A1—affect serum uric acid (SUA) levels and susceptibility of gout,^2–6^ the most common form of inflammatory arthritis. However, there are no reports on the association between *OAT10* gene and either hyperuricaemia or gout. Here, for the first time, we reveal that a dysfunctional variant of *OAT10* decreases both gout risk and SUA levels, suggesting OAT10 to be physiologically involved in urate reabsorption in the human kidney, as described below.

To explore exonic variants in *OAT10* potentially associated with gout susceptibility, we sequenced all exons of *OAT10* in 480 gout cases and 480 controls of Japanese male^6^ and conducted an association analysis (see online supplementary tables S1 and S2), followed by a replication study on 924 gout cases and 2113 controls (see online supplementary figure S1). In two identified *OAT10* variants with minor allele frequency (MAF) >0.5%, only rs117371763 (c.1129C>T; p.Arg377Cys [R377C]) was significantly associated with gout susceptibility after Bonferroni correction (p=0.014). The significant association between rs117371763 and gout susceptibility was replicated, and our meta-analysis showed a significant protective effect of rs117371763 on gout susceptibility (OR=0.67; 95% CI 0.53 to 0.85; p_meta_=7.8×10^-4^) (table 1). In addition, a quantitative trait locus analysis focusing on SUA levels in 3208 individuals (see online supplementary table S3) showed that the minor allele of rs117371763 significantly decreases SUA levels (β=–0.156 mg/dL, 95% CI –0.295 to –0.018 mg/dL, p=0.027). Results were similar even after adjustment for age.

10.1136/annrheumdis-2019-216044.supp1 Supplementary data

**Table 1:** Association analysis of *OAT10/SLC22A13* variant, rs117371763 [Arg377Cys (R377C)], with gout susceptibility

	Gout cases				Controls				p value	OR (95% CI)
	C/C	C/T	T/T	MAF (%)	C/C	C/T	T/T	MAF (%)		
Discovery phase	447	31	2	3.65	427	46	6	6.05	0.014	0.59 (0.38 to 0.90)
Replication phase	859	63	2	3.63	1900	203	5	5.02	0.015	0.71 (0.53 to 0.94)
Meta-analysis									7.8×10^-4^	0.67 (0.53 to 0.85)

In the meta-analysis, no apparent heterogeneity was observed (p value for Cochran's Q test=0.48, I^2^=0%).

MAF, minor allele frequency.

**Figure 1:**
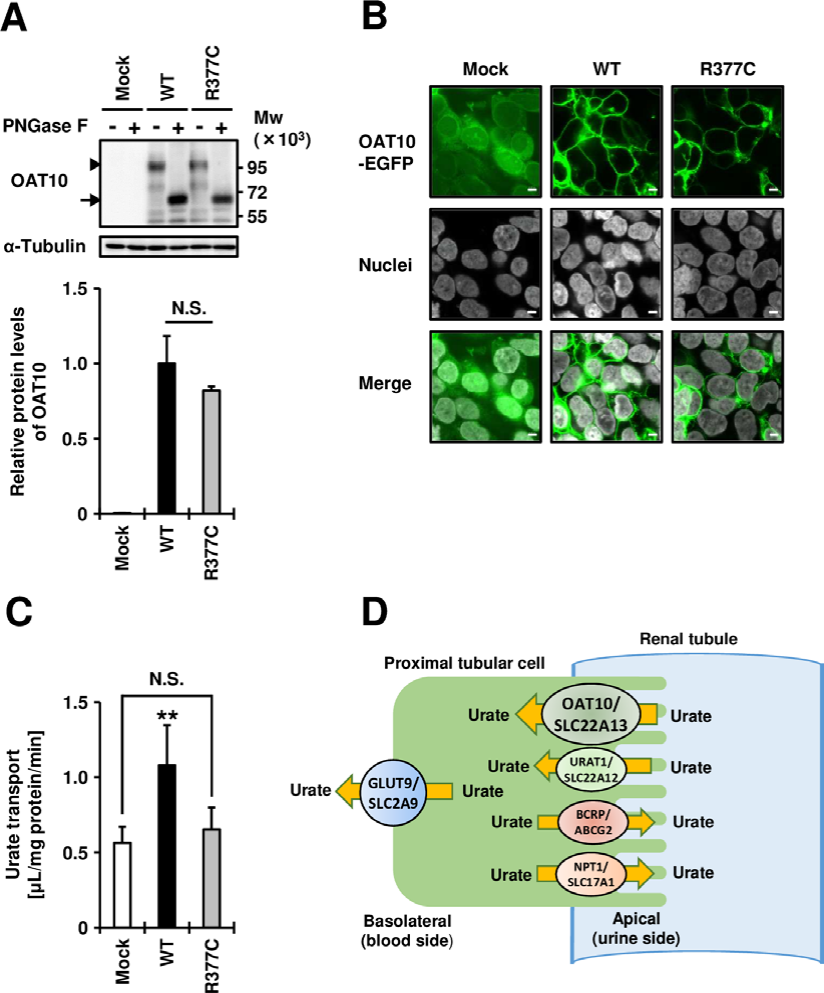
Effects of Arg377Cys (R377C) on the expression, plasma membrane localisation, and function of the organic anion transporter 10 (OAT10) urate transporter transiently expressed in 293A cells. (A) (Upper) Immunoblot detection of OAT10/SLC22A13 protein in whole cell lysate samples. OAT10 fused with EGFP was detected by an anti-EGFP antibody. Arrowhead, matured OAT10 as a glycoprotein; arrow, non-glycosylated form of OAT10; α-tubulin, a loading control; (Lower) Relative protein levels of OAT10 wild-type (WT) and Arg377Cys (R377C) variant. Data are expressed as the mean±SD, n=3. N.S., not significantly different between groups (two sided t-test). (B) Confocal microscopic observation of cellular localisation. Nuclei were stained with TO-PRO-3 iodide (grey). Bars, 5 µm. (C) Functional analysis. OAT10-expressing 293A cells were incubated with 10 µM of [^14^C]-urate for 60 s, then the amount of urate incorporated into the cells was measured. Data are expressed as the mean±SD, n=7. **p<0.01 versus the other groups (Tukey-Kramer multiple-comparison test). All experiments were performed 48 hours after plasmid transfection. (D) Proposed physiological model of OAT10 in human kidney. OAT10 is expressed on the apical membrane of renal proximal tubules and mediates reabsorption of urate from urine to blood. Other previously characterised urate reabsorption transporters (URAT1/SLC22A12 and GLUT9/SLC2A9) and urate excretion transporters (BCRP/ABCG2 and NPT1/SLC17A1) are also described.

Furthermore, *via* a series of cell-based experiments, we identified the R377C variant as an almost null variant of OAT10 (figure 1A–C). Immunoblotting and confocal microscopic observations showed the R377C variant to have little effect on OAT10 protein levels (figure 1A) or its cellular localisation (figure 1B). Cell-based urate transport assay demonstrated that, consistent with a previous report,^1^ OAT10 wild-type can transport urate (figure 1C); however, the urate transport activity of R377C variant-expressing cells was close to that of mock cells, demonstrating that this variant disrupts OAT10’s function as a urate transporter. As it is conserved across different species (see online supplementary figure S2), R377 may be important for OAT10 function.

Considering the following three points, we conclude that OAT10 is a urate reabsorption transporter on the apical side of the renal proximal tubular cells (figure 1D). First, the R377C variant of OAT10 was almost null as a urate transporter (figure 1C). Second, this dysfunctional variant decreased SUA levels (see online supplementary table S3), suggesting that functional OAT10 is physiologically involved in a supply route of urate into the blood. Third, like URAT1/SLC22A12, which plays a pivotal role in urate transport from urine to the blood,^2^ OAT10 is reportedly expressed in the brush border membranes of the renal epithelium,^1^ therefore making it a potential target for urate-lowering therapy like URAT1. Although rs117371763 of *OAT10* is common in Japanese (see online supplementary table S2), this variant is rare in other populations, including European Caucasians (see online supplementary table S4). Such populations, in which most people have functional OAT10, may offer a greater potential for OAT10 as a drug target for the treatment of gout/hyperuricaemia. Our findings will contribute to uncovering the physiological role of OAT10 as a renal urate reabsorber and its pathophysiological importance in urate-related disorders such as gout/hyperuricaemia.
