# Supplementary material for: Dysfunctional missense variant of OAT10/SLC22A13 decreases gout risk and serum uric acid levels
Source: Ann Rheum Dis. 2019 Nov 28;79(1):164–6. doi: 10.1136/annrheumdis-2019-216044 (PMC6937405; doi:10.1136/annrheumdis-2019-216044)
Supplement: Supplementary data [file annrheumdis-2019-216044supp001.pdf]

Supplementary Materials

Dysfunctional missense variant of *OAT10/SLC22A13* decreases gout risk and serum uric acid levels

T. Higashino, K. Morimoto, H. Nakaoka, Y. Toyoda, Y. Kawamura and S. Shimizu *et al*.
Correspondence to H. Matsuo (email: hmatsuo@ndmc.ac.jp) and T. Takada (email: tappei-tky@umin.ac.jp).

Supplementary Methods
Supplementary References
Supplementary Figure S1: Study design
Supplementary Figure S2: Two-dimensional structural insight of Arg377Cys (R377C) variant of OAT10 protein
Supplementary Table S1: Characteristics of the participants
Supplementary Table S2: Nonsynonymous variants of *OAT10/SLC22A13* identified in the discovery phase of association analysis
Supplementary Table S3: Quantitative trait locus analysis of OAT10/SLC22A13 variant, rs117371763 (1129C>T, R377C), on serum uric acid levels
Supplementary Table S4: Allele frequencies of *OAT10/SLC22A13* variant, rs117371763, in each population
Supplementary Table S5: Key resources for cell-based molecular analyses

Higashino T, *et al*. *Ann Rheum Dis* 2019; 79:1–2. doi: 10.1136/annrheumdis-2019-216044

Supplementary Methods

Patients and controls

This study was approved by the institutions' Ethical Committees (National Defense Medical College, National Institute of Genetics, and Nagoya University). All protocols were in accordance with the Declaration of Helsinki, and written informed consent was obtained from all the participants.

We recruited 1404 gout patients from the Ryougoku East Gate Clinic and Midorigaoka Hospital. All patients were clinically diagnosed as primary gout with the criteria of American College of Rheumatology[1]. Patients with inherited metabolic disorders, including Lesch–Nyhan syndrome, were excluded from this study. As controls, 3208 individuals without the history of gout or urate-lowering therapy (ULT) were recruited from health examinees at Self-Defense Forces Central Hospital, and the participants of Shizuoka study and Daiko study, which belong to the Japan Multi-Institutional Collaborative Cohort (J-MICC) Study[2]. They were classified into 2593 "normouricemic controls" (SUA levels ≤ 7.0 mg/dL) and 615 "hyperuricemic controls" (SUA levels > 7.0 mg/dL).

We analyzed 480 gout patients and 480 normouricemic controls, which were collected in our previous study[3], in the discovery phase of the association analysis: 924 gout patients and 2113 normouricemic controls in the replication phase (**Supplementary Figure S1**). QTL analysis of SUA was performed for 3208 Japanese individuals as controls (**Supplementary Figure S1**). The characteristics of the participants are shown in **Supplementary Table S1**.

Genetic and statistical analyses of human OAT10/SLC22A13 gene

Genomic DNA was extracted from the whole peripheral blood cells of the participants[4]. In the discovery phase, to explore potential candidates of *OAT10* variants that can affect gout susceptibility, we performed targeted exon sequencing of *OAT10* as described previously[3,5]. Briefly, 20 ng of DNA was

fragmented and adapter-ligated using a SureSelect QXT Library Prep Kit (Agilent Technologies, Santa Clara, CA, USA). The fragmented libraries with distinct indexed adapters were pooled at equimolar amounts. Target enrichment was then conducted using the SeqCap EZ Choice System (Roche Diagnostics, Tokyo, Japan). A DNA probe set complementary to *OAT10* was selected using NimbleDesign (https://design.nimblegen.com). The libraries were sequenced on an Illumina HiSeq 2500 platform with a 2×100-bp paired-end module (Illumina, San Diego, CA, USA). As the quality control step, the reads containing the Illumina adapter sequences and low quality sequences were trimmed using Trimmomatic[6]. The sequence reads were aligned to human reference genome (hg19) using BWA[7]. The aligned reads were processed for removal of PCR duplicates using Picard tools (https://broadinstitute.github.io/picard/), and for local realignment and base quality recalibration by GATK[8]. Single nucleotide variants (SNVs) and insertions and deletions were detected using GATK's HaplotypeCaller[8]. Functional annotation of the identified variants was implemented by ANNOVAR[9]. After variant calling and annotation, we selected missense and nonsense SNVs as well as insertions and deletions in the exons of *OAT10* based on the DNA reference sequence NM_004256; we excluded synonymous SNVs in *OAT10* exons as well as variants in introns or untranslated regions for the downstream association analysis. In the replication phase of the association analysis, rs117371763 (1129C>T: p. Arg377Cys [R377C]) of *OAT10* was genotyped using a TaqMan method (ThermoFisher Scientific Inc., MA, USA) with a LightCycler 480 (Roche Diagnostics, Mannheim, Germany) as previously described[10]. In a quantitative trait locus (QTL) analysis of SUA, we used genotyping data of normouricemic controls obtained in both discovery and replication phases of the association analysis. Additionally, we genotyped rs117371763 of hyperuricemic controls with the TaqMan method[10]. To confirm rs117371763 genotypes, >100 samples were subjected to direct sequencing. Direct sequencing was performed with the following primers: forward 5′-TGGTGTGTGTTGGCAGAG-3′ and reverse 5′-GGTCCCATCCACTGGAAC-3′. DNA sequence analysis was performed with a 3130xl Genetic

Analyzer (ThermoFisher Scientific) [10]. Statistical analyses were performed using SPSS v.22.0J (IBM Japan Inc., Tokyo, Japan) and the software R (version 3.1.1) with meta package. The $\chi 2$ test was used for association analyses; the univariate linear regression analysis was used for the QTL analysis. In the discovery phase, we chose nonsynonymous *OAT10* variants with minor allele frequency (MAF) greater than 0.5% either in cases or controls and analyzed their association with gout susceptibility. After the replication phase, we also performed a meta-analysis using the DerSimonian and Laird random-effects model. Cochran's Q test and I^2 were used for the evaluation of heterogeneity between two phases of the association analysis. A p value less than 0.05 was considered statistically significant. In addition, this statistical threshold was adjusted by Bonferroni correction accordingly.

Materials for all based molecular analyses

All chemicals used were commercially available and were of analytical grade. Critical materials used are summarized in **Supplementary Table S5**.

Construction of OAT10-containing expression vector

As a sub-cloning source, an I.M.A.G.E Full length cDNA clone (GenBank: BC035973.1) was purchased from K.K. DNAFORM (Kanagawa, Japan). We corrected some mutations in the clone using a site-directed mutagenesis technique, and then we successfully obtained the full-length human OAT10 wild-type (WT) (NCBI accession; NM_004256) open reading frame (ORF). For CMV-driven expression of OAT10-EGFP in mammalian cells, the OAT10 ORF was inserted into the pEGFP-N1 vector plasmid (Clontech Laboratories, Inc., Palo Alto, CA, USA) according to our previous study[11]. Using the site-directed mutagenesis technique, an original stop codon of OAT10 was removed and a mutant of OAT10 harboring rs117371763 (1129C>T: p.R377C) was constructed using the pEGFP-N1 vector. Introduction of the mutation was confirmed by direct sequencing using BigDye® Terminator v3.1

(Applied Biosystems Inc., Foster City, CA, USA) with Applied Biosystems® 3130 Genetic Analyzer (Applied Biosystems Inc.) according to the manufacturer's protocol.

Cell culture

Human embryonic kidney 293 (HEK293)-derived 293A cells were maintained in Dulbecco's modified Eagle's medium (Nacalai Tesque, Inc., Kyoto, Japan) supplemented with 10% fetal bovine serum (Biowest, Nuaillé, France), 1% penicillin-streptomycin (Nacalai Tesque), 2 mM L-glutamine (Nacalai Tesque), and 1 × non-essential amino acid (ThermoFisher Scientific) at 37ºC in a humidified atmosphere of 5% (v/v) CO_2 in air.

Each vector plasmid for OAT10 or mock (pEGFP-N1) was transfected into 293A cells, using polyethyleneimine "MAX" (PEI-MAX) (Polysciences Inc., Warrington, PA, USA) as described previously[12], followed by 48-hour incubation before a cell-based transport assay.

Preparation of protein lysates and immunoblotting

Whole cell lysates were prepared with cell lysis buffer A containing 50 mM Tris/HCl (pH 7.4), 1 mM dithiothreitol, 1% (w/v) Triton X-100, and a protease inhibitor cOmplete, EDTA free (Roche, Basel, Switzerland), followed by treatment with Peptide *N*-glycosidase F (PNGase F) (New England Biolabs, Inc., Ipswich, MA, USA) as described previously[11]. The protein concentration was determined using the Pierce™ BCA Protein Assay Kit (Thermo Fisher Scientific) with BSA as a standard according to the manufacturer's protocol.

Immunoblotting was performed according to our previous study[3]. Blots were probed with appropriate antibodies (**Supplementary Table S5)**, and then the signals were visualized by a chemiluminescent method. All antibodies were used at 1:1000 (first antibody) or 1:2000 (second antibody) dilution. After washing with Tris-buffered saline containing 0.05% Tween 20 for 1 hour at

room temperature, HRP-dependent luminescence was developed with ECL™ Prime Western Blotting Detection Reagent (GE Healthcare UK Ltd., Buckinghamshire, UK) and detected using a luminescent image analyzer (Bio-Rad Laboratories, Tokyo, Japan). For the quantification of OAT10 protein levels, the signal intensity ratio (OAT10/α-tubulin) of the immunoreactive bands corresponding to the non-glycosylated form of OAT10 after PNGase F treatment was determined and normalized with that in OAT10 WT-expressing cells.

Confocal microscopic observation

Forty-eight hours after transfection, 293A cells were fixed with ice cold methanol and subjected to TO-PRO-3 Iodide (Molecular Probes, Eugene, OR, USA) staining as described previously[13]. After the visualization of nuclei, the cells mounted in VECTASHIELD Mounting Medium (Vector Laboratories, Burlingame, CA, USA) were observed using an FV10i Confocal Laser Scanning Microscope (Olympus, Tokyo, Japan). The obtained data were analyzed using an Imaris software (Carl Zeiss Japan, Tokyo, Japan).

Cell-based transport assay using OAT10-expressing 293A cells

The urate uptake assay using OAT10-expressing 293A cells was conducted according to a previous study[14] with some modifications. In brief, 48 hours after the plasmid transfection, the cells were washed twice with a transport buffer (TP-Buffer: 130 mM NaCl, 4 mM KCl, 1 mM Na_2HPO_4, 1 mM $MgSO_4$, 1 mM $CaCl_2$, 20 mM HEPES, 18 mM D-glucose, and pH 7.4) and pre-incubated in TP-Buffer for 15 minutes at 37°C. Then, the buffer was exchanged with a pre-warmed fresh TP-Buffer containing 10 μM [8-^{14}C]-urate, and the cells were further incubated for 60 sec. Then, the cells were washed with ice-cold TP-Buffer twice and dissolved in 0.2N NaOH according to our previous study[12]. After neutralization with 1N HCl, the radioactivity in the lysate was measured using a liquid scintillator (Tri-Carb 3110TR, PerkinElmer, Inc., Waltham, MA, USA). The protein concentrations were determined using the Pierce™

Higashino T, *et al. Ann Rheum Dis* 2019; 79:1–2. doi: 10.1136/annrheumdis-2019-216044

BCA Protein Assay Kit. The urate transport activity was calculated as the incorporated clearance (μL/mg protein/min): (incorporated level of urate [DPM/mg protein/min]/urate level in the incubation mixture [DPM/μL]).

Quantification and statistical analysis for cell-based molecular analyses

All statistical analyses were performed using Excel 2013 (Microsoft Corp., Redmond, WA, USA) with Statcel3 add-in software (OMS publishing Inc., Saitama, Japan). Different statistical tests were used for different experiments as described in the figure legends. Briefly, when analyzing multiple groups, the similarity of variance between groups was compared using Bartlett's test. When passing the test for homogeneity of variance, a parametric Dunnett's test was used. In the case of a single pair of quantitative data, after comparing the variances of a set of data by *F*-test, unpaired Student's *t*-test was performed. Statistical significance was defined in terms of p values less than 0.05 or 0.01.

Supplementary References

1. Wallace SL, Robinson H, Masi AT, *et al.* Preliminary criteria for the classification of the acute arthritis of primary gout. *Arthritis Rheum* 1977;20:895-900.
2. Hamajima N, Group JMS. The Japan Multi-Institutional Collaborative Cohort Study (J-MICC Study) to detect gene-environment interactions for cancer. *Asian Pac J Cancer Prev* 2007;8:317-23.
3. Higashino T, Takada T, Nakaoka H, *et al.* Multiple common and rare variants of ABCG2 cause gout. *RMD Open* 2017;3:e000464.
4. Matsuo H, Chiba T, Nagamori S, *et al.* Mutations in glucose transporter 9 gene SLC2A9 cause renal hypouricemia. *Am J Hum Genet* 2008;83:744-51.
5. Ahmadloo S, Nakaoka H, Hayano T, *et al.* Rapid and cost-effective high-throughput sequencing for identification of germline mutations of BRCA1 and BRCA2. *J Hum Genet* 2017;62:561-67.
6. Bolger AM, Lohse M, Usadel B. Trimmomatic: a flexible trimmer for Illumina sequence data. *Bioinformatics* 2014;30:2114-20.
7. Li H, Durbin R. Fast and accurate short read alignment with Burrows-Wheeler transform. *Bioinformatics* 2009;25:1754-60.
8. DePristo MA, Banks E, Poplin R, *et al.* A framework for variation discovery and genotyping using

Higashino T, *et al. Ann Rheum Dis* 2019; 79:1–2. doi: 10.1136/annrheumdis-2019-216044

next-generation DNA sequencing data. *Nat Genet* 2011;43:491-8.
9. Wang K, Li M, Hakonarson H. ANNOVAR: functional annotation of genetic variants from high-throughput sequencing data. *Nucleic Acids Res* 2010;38:e164.
10. Sakiyama M, Matsuo H, Shimizu S*, et al.* The effects of URAT1/SLC22A12 nonfunctional variants, R90H and W258X, on serum uric acid levels and gout/hyperuricemia progression. *Sci Rep* 2016;6:20148.
11. Toyoda Y, Takada T, Miyata H*, et al.* Regulation of the Axillary Osmidrosis-Associated ABCC11 Protein Stability by N-Linked Glycosylation: Effect of Glucose Condition. *PLoS One* 2016;11:e0157172.
12. Miyata H, Takada T, Toyoda Y*, et al.* Identification of Febuxostat as a New Strong ABCG2 Inhibitor: Potential Applications and Risks in Clinical Situations. *Front Pharmacol* 2016;7:518.
13. Toyoda Y, Mancikova A, Krylov V*, et al.* Functional Characterization of Clinically-Relevant Rare Variants in ABCG2 Identified in a Gout and Hyperuricemia Cohort. *Cells* 2019;8
14. Bahn A, Hagos Y, Reuter S*, et al.* Identification of a new urate and high affinity nicotinate transporter, hOAT10 (SLC22A13). *J Biol Chem* 2008;283:16332-41.

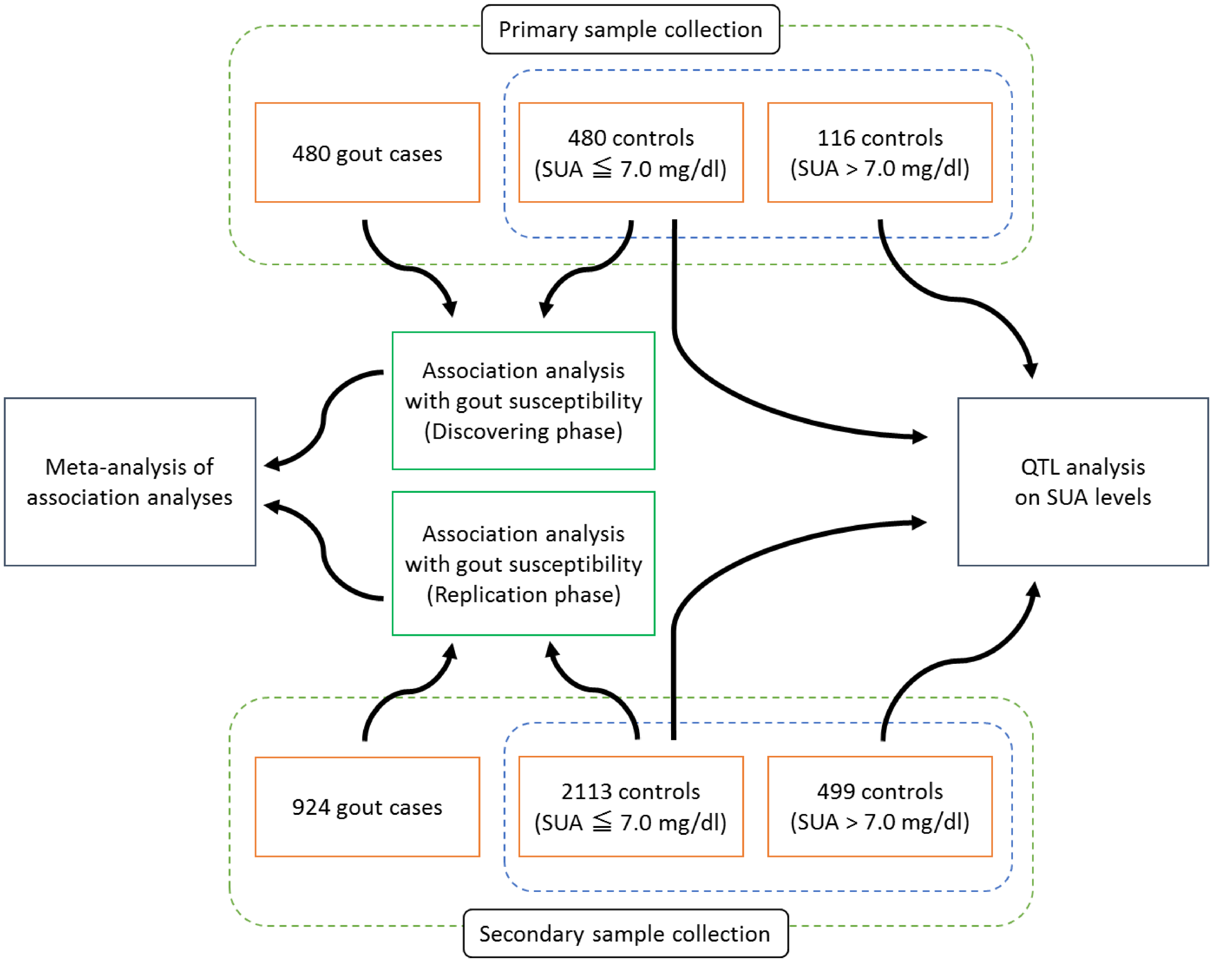

Supplementary Figure S1　Study Design.

We recruited 1404 gout patients. As controls, 3208 individuals without the history of gout or urate-lowering therapy were recruited and classified into 2593 “normouricemic controls” (SUA levels ≤ 7.0 mg/dL) and 615 “hyperuricemic controls” (SUA levels > 7.0 mg/dL). We analyzed 480 gout patients and 480 normouricemic controls in the discovery phase of the association analysis: 924 gout patients and 2113 normouricemic controls in the replication phase. QTL analysis on SUA levels was performed for 3208 Japanese individuals as controls.

QTL, quantitative trait locus; SUA, serum uric acid

Higashino T, *et al. Ann Rheum Dis* 2019; 79:1–2. doi: 10.1136/annrheumdis-2019-216044

A

	Arg377Cys	
Homo sapiens	370 G A V E V P A R C S S I F M M 384	NP_004247
Pan troglodytes	G A V E V P A R C S S I F M M	XP_526175
Macaca mulatta	G A V E V P A R C S S I F M M	XP_001087330
Rattus novergicus	G A V E M P G R F L S V L M M	NP_001119757
Mus musculus	G V V E V P A R L S S I P M M	NP_598741

B

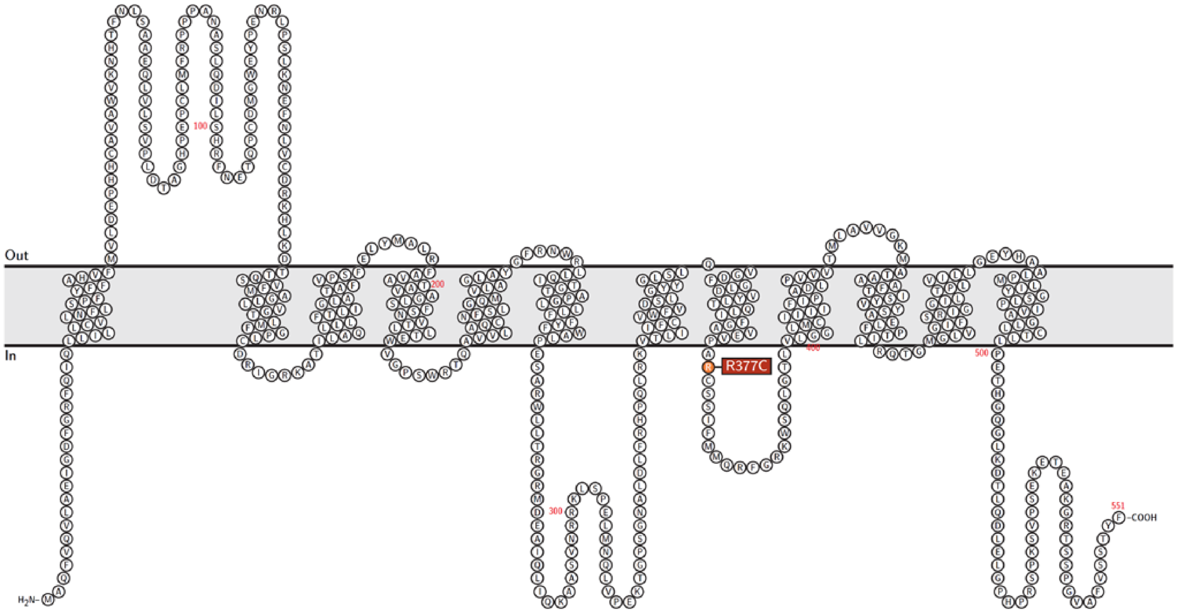

Supplementary Figure S2 Two-dimensional structural insight of Arg377Cys (R377C) variant of OAT10 protein.

(A) OAT10 amino acids evolutionarily conserved across five mammalian species. The position of the amino acid at 377 is labelled in grey. Multiple sequence alignments and homology calculations were carried out using Genetyx software (Genetyx Co., Tokyo, Japan) with the ClustalW2.1 Windows program. (B) Schematic illustration of a putative topological model of human OAT10 protein. A 2D topology of OAT10 protein was constructed using the Protter program (http://wlab.ethz.ch/protter/start/), and the obtained topology data were plotted and modified using the T(E)Xtopo package.

Supplementary Table S1 Characteristics of the participants*

	Discovery phase		Replication phase		QTL analysis
	**Case**	**Control**	**Case**	**Control**	**on SUA**
Number	480	480	924	2113	3208
Call rate (%)	100	99.8	100	99.8	99.8
Age (year)	46.2 ± 9.8	53.0 ± 7.9	46.1 ± 11.0	51.5 ± 7.2	51.8 ± 7.3
Body-mass index (kg/m^2)	25.3 ± 3.7	23.2 ± 2.6	25.2 ± 3.6	23.7 ± 3.1	23.8 ± 3.1
SUA (mg/dl)	8.40 ± 1.14	5.70 ± 0.87	8.45 ± 1.24	5.72 ± 1.00	6.12 ± 1.24

*All participants were Japanese male.

Plus-minus values are means ± standard deviation.

QTL, quantitative trait locus; SUA, serum uric acid

Supplementary Table S2 Nonsynonymous variants of *OAT10/SLC22A13* identified in the discovery phase of association analysis

rs number	Position*	Change in DNA sequence†	AA change	MAF (%)		p Value	OR (95% CI)
				Cases	Controls		
N/A	38307587	A236C	H79P	1.68	0.941	0.16	1.8 (0.79 to 4.1)
rs117371763	38317479	C1129T	R377C	3.65	6.05	0.014	0.59 (0.38 to 0.90)

Nonsynonymous variants with minor allele frequency >0.5% in either gout cases or controls are shown in this table.

*Positions refer to the GRCh37 assembly.

†Nucleotide numbering is based on the DNA reference sequence NM_004256.

AA, amino acid; MAF, minor allele frequency; OR, odds ratio; CI, confident interval; N/A, not applicable

We considered that a raw p value less than 0.025 indicates statistical significance after Bonferroni correction.

Higashino T, *et al. Ann Rheum Dis* 2019; 79:1–2. doi: 10.1136/annrheumdis-2019-216044

Supplementary Table S3 Quantitative trait locus analysis of *OAT10/SLC22A13* variant, rs117371763 (1129C>T, R377C), on serum uric acid levels

	Genotypes			Crude		Age-adjusted	
	**C/C**	**C/T**	**T/T**	**p Value**	β (95%CI)	**p Value**	β (95%CI)
Number	2900	288	12	0.027	−0.156 (−0.295 to −0.018)	0.024	−0.160 (−0.298 to −0.021)
SUA (mg/dl)	6.13 ± 1.25	5.98 ± 1.11	5.83 ± 1.24				

SUA values are mean ± standard deviation.

β is for per copy of the allele,

CI, confidence interval; SUA, serum uric acid

Supplementary Table S4 Allele frequencies of *OAT10/SLC22A13* variant, rs117371763, in each population

Population	Ref	Alt	MAF (%)	Database (version)	Identifier
Japanese	6087	311	4.86	Present study*	
Japanese	2210	94	4.08	Human Genetic Variation Database (v2.3)	http://www.hgvd.genome.med.kyoto-u.ac.jp
Japanese	6719	299	4.26	Integrative Japanese Genome Variation Database (3.5KJPNv2)	https://ijgvd.megabank.tohoku.ac.jp
Korean	207	1	0.481	Korean Personal Genome Project	http://opengenome.net/index.php/Main_Page
East Asian	997	11	1.09	1000 Genomes Project Phase 3	http://www.ensembl.org
Chinese Dai in Xishuangbanna	186	0	0	1000 Genomes Project Phase 3	http://www.ensembl.org
Han Chinese in Beijing	206	0	0	1000 Genomes Project Phase 3	http://www.ensembl.org
Southern Han Chinese	210	0	0	1000 Genomes Project Phase 3	http://www.ensembl.org
Japanese in Tokyo	197	11	5.28	1000 Genomes Project Phase 3	http://www.ensembl.org
Kinh in Ho Chi Minh City	198	0	0	1000 Genomes Project Phase 3	http://www.ensembl.org
African	1322	0	0	1000 Genomes Project Phase 3	http://www.ensembl.org
American	694	0	0	1000 Genomes Project Phase 3	http://www.ensembl.org
European	999	1	0.100	1000 Genomes Project Phase 3	http://www.ensembl.org
South Asian	978	0	0	1000 Genomes Project Phase 3	http://www.ensembl.org

*The sample set used for the quantitative trait locus analysis

Ref, number of reference allele (C); Alt, number of alternative allele (T); MAF, minor allele frequency

Higashino T, *et al. Ann Rheum Dis* 2019; 79:1–2. doi: 10.1136/annrheumdis-2019-216044

Supplementary Table S5 Key resources for cell-based molecular analyses

Reagent or Resource	Source	Identifier
Antibodies		
Rabbit polyclonal anti-EGFP	Life technologies	Cat# A11122; RRID:AB_221569
Rabbit polyclonal anti-α-tubulin	Abcam	Cat# ab15246; RRID: AB_301787
Donkey anti-rabbit IgG-horseradish peroxidase (HRP)-conjugate	GE Healthcare	Cat# NA934V; RRID:AB_772206
Chemicals		
[8-^{14}C]-uric acid (53 mCi/mmol)	American Radiolabeled Chemicals Inc.	Cat# ARC0513
Polyethelenimine “MAX” (PEI-MAX)	Polysciences	Cat# 24765; CAS: 49553-93-7
Critical Commercial Assays		
Pierce™ BCA Protein Assay Reagent A	Thermo Fisher Scientific	Cat# 23223
Pierce™ BCA Protein Assay Reagent B	Thermo Fisher Scientific	Cat# 23224
PureLink™ HiPure Plasmid Filter Midiprep Kit	Thermo Fisher Scientific	Cat# K210015
Recombinant DNA		
The complete human OAT10 cDNA	This paper	NCBI Reference Sequence: NM_004256
Experimental Models: Cell Lines		
293A	Invitrogen	R70507
Software and Algorithms		
Excel 2013	Microsoft Corp.	https://products.office.com/ja-jp/microsoft-excel-2013
Statcel3 add-in software	OMS publishing	http://www.oms-publ.co.jp/
Imaris	Carl Zeiss Japan	https://www.zeiss.co.jp/microscopy/local/local-products/imaris.html

Higashino T, *et al. Ann Rheum Dis* 2019; 79:1–2. doi: 10.1136/annrheumdis-2019-216044
